# The Prevalence of Cam Morphology: A Cross-Sectional Evaluation of 3,558 Cadaveric Femora

**DOI:** 10.3389/fsurg.2020.588535

**Published:** 2021-01-21

**Authors:** Shane Hanzlik, Andrew J. Riff, Thomas H. Wuerz, Michael Abdulian, Danielle Gurin, Shane J. Nho, Michael J. Salata

**Affiliations:** ^1^Department of Orthopaedics, UH Case Medical Center, Cleveland, OH, United States; ^2^Rush University Medical Center, Midwest Orthopaedics at Rush, Chicago, IL, United States; ^3^Sports and Orthopaedic Specialists, Edina, MN, United States; ^4^Glendale Orthopaedics, Glendale, CA, United States; ^5^Cleveland Clinic South Pointe, Warrensville Heights, OH, United States

**Keywords:** hip, FAI, femoroacetabular impingement, hip arthroscopy, cadaveric

## Abstract

**Purpose:** We sought to determine (1) the prevalence of cam deformity in the population and that of bilateral cam deformity, (2) the typical location of a cam lesion, and (3) the typical size of a cam lesion by direct visualization in cadaveric femora.

**Methods:** Two observers inspected 3,558 human cadaveric femora from the Hamann–Todd Osteological Collection from the Cleveland Museum of Natural History. Any asphericity >2 mm from the anterior femoral neck line was classified as a cam lesion. Once lesions had been inspected, the prevalence in the population, prevalence by gender, and prevalence of bilateral deformity were determined. Additionally, each lesion was measured and localized to a specific quadrant on the femoral neck based upon location of maximal deformity.

**Results:** Cam lesions were noted in 33% of males and 20% of females. Eighty percent of patients with a cam lesion had bilateral lesions. When stratified by location of maximal deformity, 90.9% of lesions were in the anterosuperior quadrant and 9.1% were in the anteroinferior quadrants. The average lesion measured 17 mm long × 24 mm wide × 6 mm thick in men and 14 mm × 22 mm × 4 mm in women (*p* < 0.05).

**Conclusions:** The population prevalence of cam deformity determined by direct visualization in cadavers may be higher than has been suggested in studies utilizing imaging modalities.

**Level of Evidence** : Level II, diagnostic study.

## Introduction

Femoroacetabular impingement (FAI) is a pathologic entity defined by abnormal contact forces in the hip joint secondary to morphologic changes involving the femoral head–neck junction or acetabulum rim. This abnormal contact has been implicated in the development of labral tears, chondral lesions, and early osteoarthritis (1). As much as 40–50% of degenerative osteoarthritis of the hip is thought to occur due to FAI (2, 3). FAI has been categorized into three different subtypes: cam impingement, pincer impingement, and combined cam and pincer impingement (4).

Isolated cam impingement refers to FAI resulting from abnormal morphology of the proximal femur including asphericity of the femoral head, decreased head–neck ratio, and femoral neck retroversion. Numerous etiologies have been proposed including genetic factors, activity-related factors (e.g., basketball, football, and hockey), and history of pediatric hip disease (e.g., slipped capital femoral epiphysis or Legg–Calve–Perthes disease) (5). Regardless of the etiology, the presence of cam morphology alone confers an increased risk of osteoarthritis of the hip (6, 7). Gosvig et al. (8) demonstrated that an aspherical femoral head [defined by elevated alpha angle (>83 in men or >57 in women) or elevated triangular index (≥0)] resulted in a 2.2-fold increase in a patient's risk of developing osteoarthritis after controlling for age, gender, and other morphologic abnormalities. Likewise, Agricola et al. (7) demonstrated in a prospective cohort study of over 1,400 hips that moderate (α angle >60°) and severe (α angle >83°) cam morphology on anteroposterior (AP) pelvis radiograph resulted in a significantly higher risk for developing end-stage osteoarthritis. Bardakos and Villar (9) and Clohisy et al. (10) independently evaluated cohorts of 43 and 70 patients with cam deformity and noted progression to osteoarthritis in 65 and 73%, respectively. In light of the correlation between cam deformity and hip osteoarthritis, the clinical and epidemiological burden of disease of hip osteoarthritis, and its economic societal impact, it is valuable to know the prevalence of cam lesions in the North American population. Several studies have evaluated the prevalence of cam impingement in the asymptomatic adult hip using plain radiographs, CT, and MRI, arriving at rates ranging between 14 and 25% in men and 4 and 17% in women (11–15). However, each of these radiographic modalities has been questioned with regard to its accuracy and its interobserver reliability (16, 17).

The purpose of our study was to determine (1) the prevalence of cam lesions in a large-scale regional sample of the Cleveland metropolitan area, (2) the proportion of cam lesions found in each quadrant, and (3) the typical size of a cam lesion by direct visualization in cadaveric femora. We hypothesized that the prevalence of cam deformity identified in our sample using direct inspection would be greater than that reported elsewhere in the literature in studies using imaging modalities.

## Methods

Two orthopedic surgery residents inspected 3,558 human cadaveric femora (from 1,907 individuals) from the Hamann–Todd Osteological Collection at the Cleveland Museum of Natural History. The Hamann–Todd collection consists of disarticulated human skeletons of 3,592 humans gathered from regional Cleveland hospitals and morgues between 1912 and the 1938 (18). We included any femur available in the collection aged 18–55. We excluded any femora with obvious post-traumatic deformity, full-thickness cartilage eburnation, or significant osteophytes at the margin of the femoral head.

**Figure 1:**
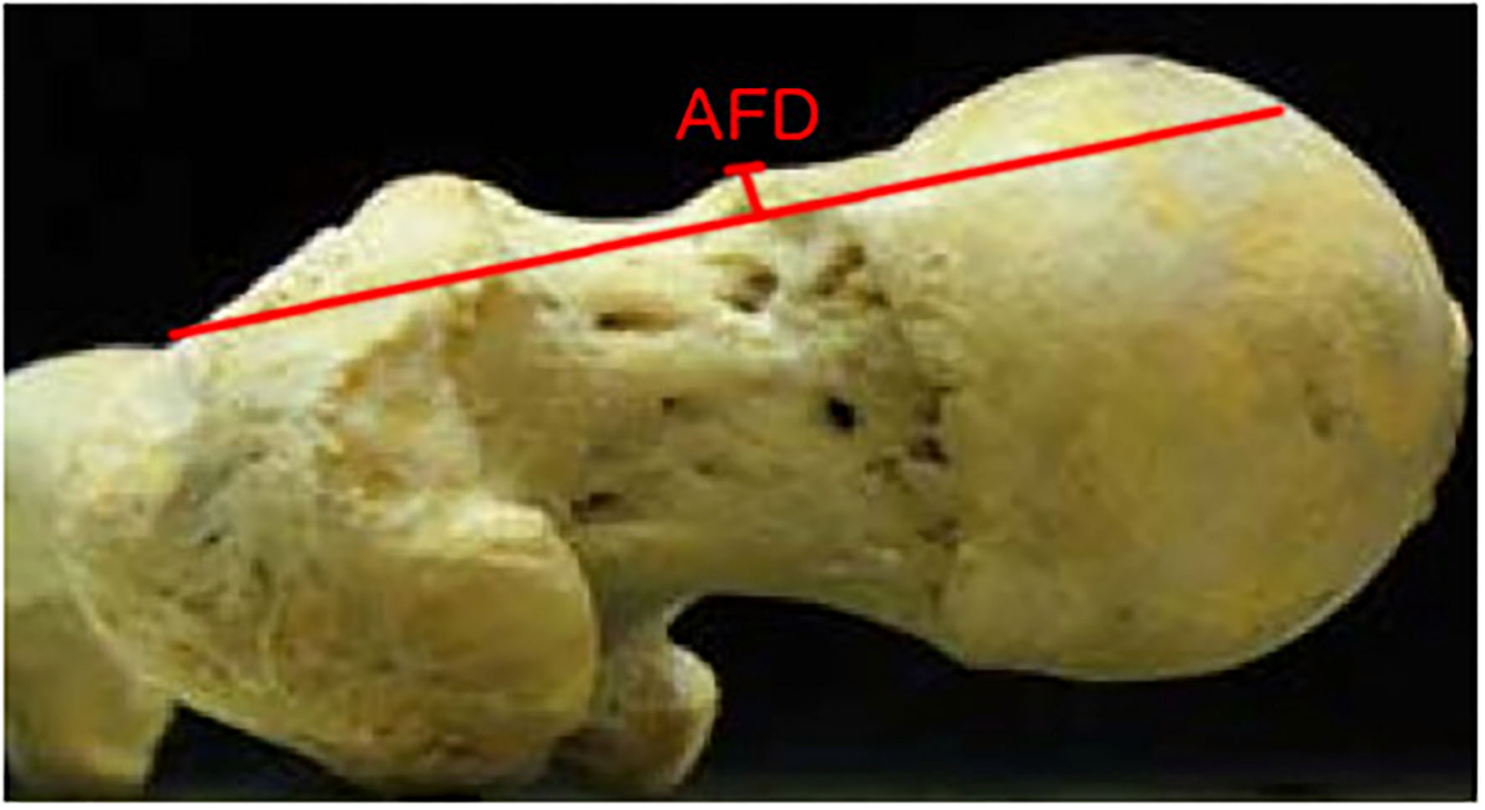
Axial view of a cadaveric femur. The anterior femoral distance (AFD) as described by Lohan et al. (17) is the greatest perpendicular depth of epiphyseal overgrowth at the head–neck junction measured from the anterior femoral line.

**Figure 2:**
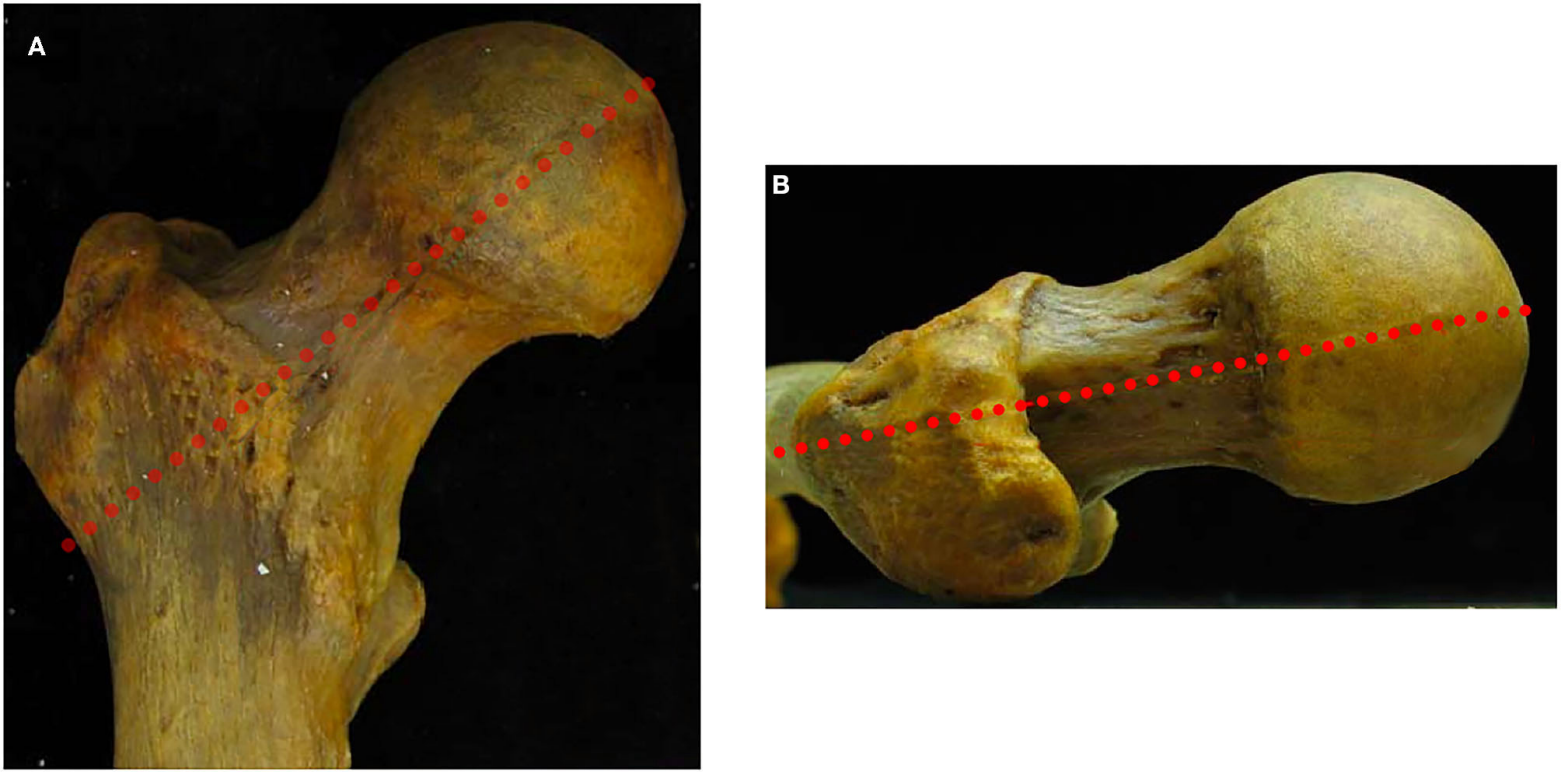
Coronal (**A**) and axial views (**B**) of a cadaveric femur demonstrating the manner in which the femoral neck was divided into superior/inferior and anterior/posterior halves.

Using visual inspection, we first identified those femora with a noticeable asphericity at the head–neck junction. Any visible asphericity >2 mm from the anterior femoral neck line [i.e., anterior femoral distance (AFD) >2 mm, described by Lohan et al. (17)] was identified as a cam lesion (Figure 1). The femoral neck was divided into anterior and posterior halves and superior and inferior halves to define four quadrants. The lines dividing the anterior and posterior halves and superior and inferior halves were both parallel to the axis of the femoral neck through the center of the femoral head (Figures 2A,B). When a cam lesion was identified, the lesion was assigned to one of four quadrants based on where the largest portion of the cam was located.

**Figure 3:**
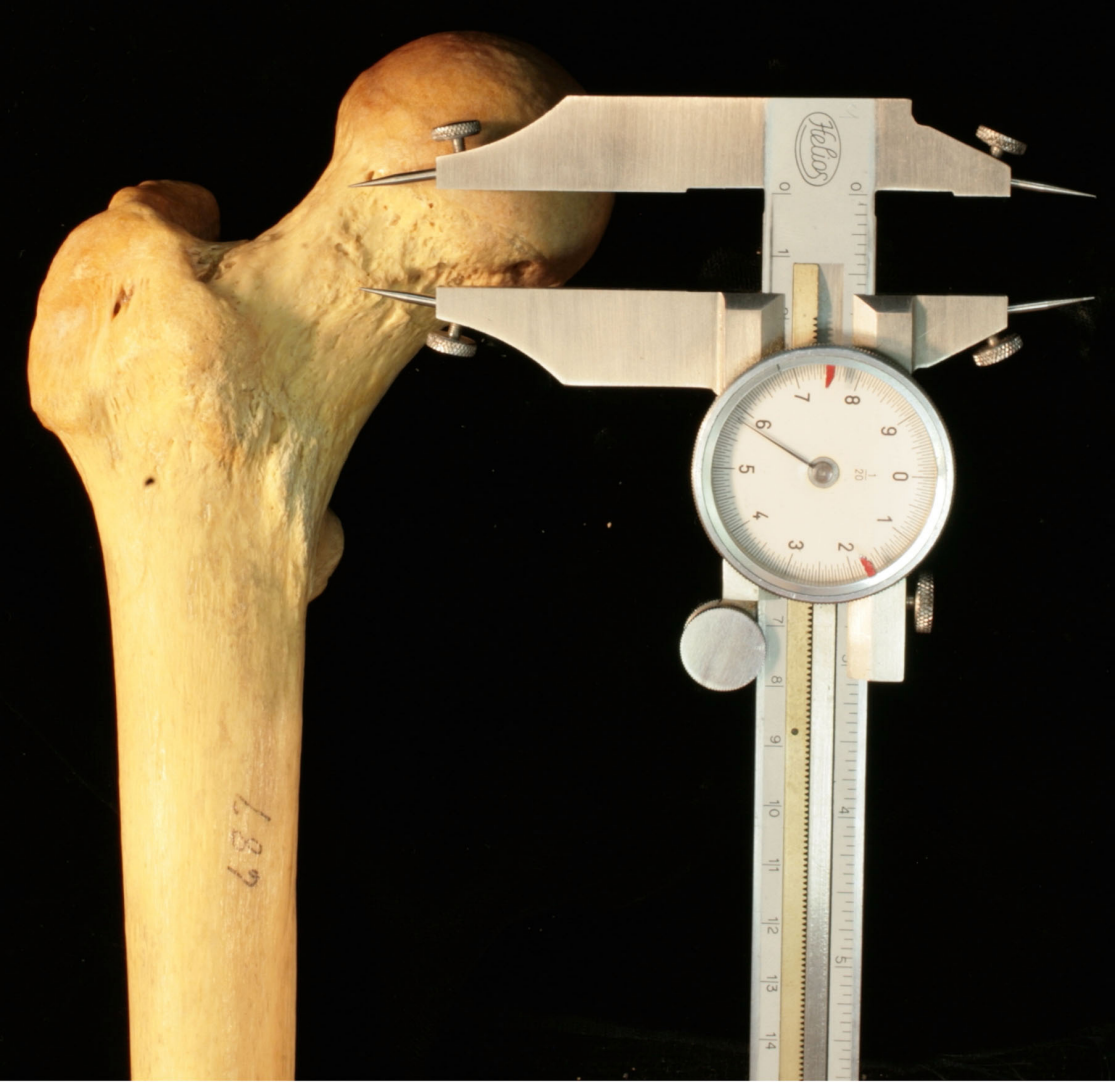
Coronal view of a cadaveric femur demonstrating the use of digital calipers used to measure the length of a cam lesion from superior to anterior.

The cam lesion was measured in three dimensions using handheld digital calipers including length, width, and thickness. Length was measured from the superior border of the cam deformity to the most inferior portion, as displayed in Figure 3. The width measurement consisted of measuring the cam deformity from the most medial to the most lateral edge. Thickness was a measurement from anterior to posterior. To measure the thickness of the lesion, we measured the AFD, as used by Lohan et al. (17), in which the femoral neck was visualized using an axial view (Figure 1). The size of the cam lesion was then measured in millimeters as the distance the bump was elevated from the anterior neck line. A Student's *T*-test with significance level of 0.05 was used to determine the difference in size between lesions in men and women and right and left lower extremities.

To measure intraobserver and interobserver reliability, we randomly chose 20 of the cadaveric femora and each femur was measured twice by each of two independent observers in two alternate random orders.

## Results

### Overall Prevalence

**Figure 4:**
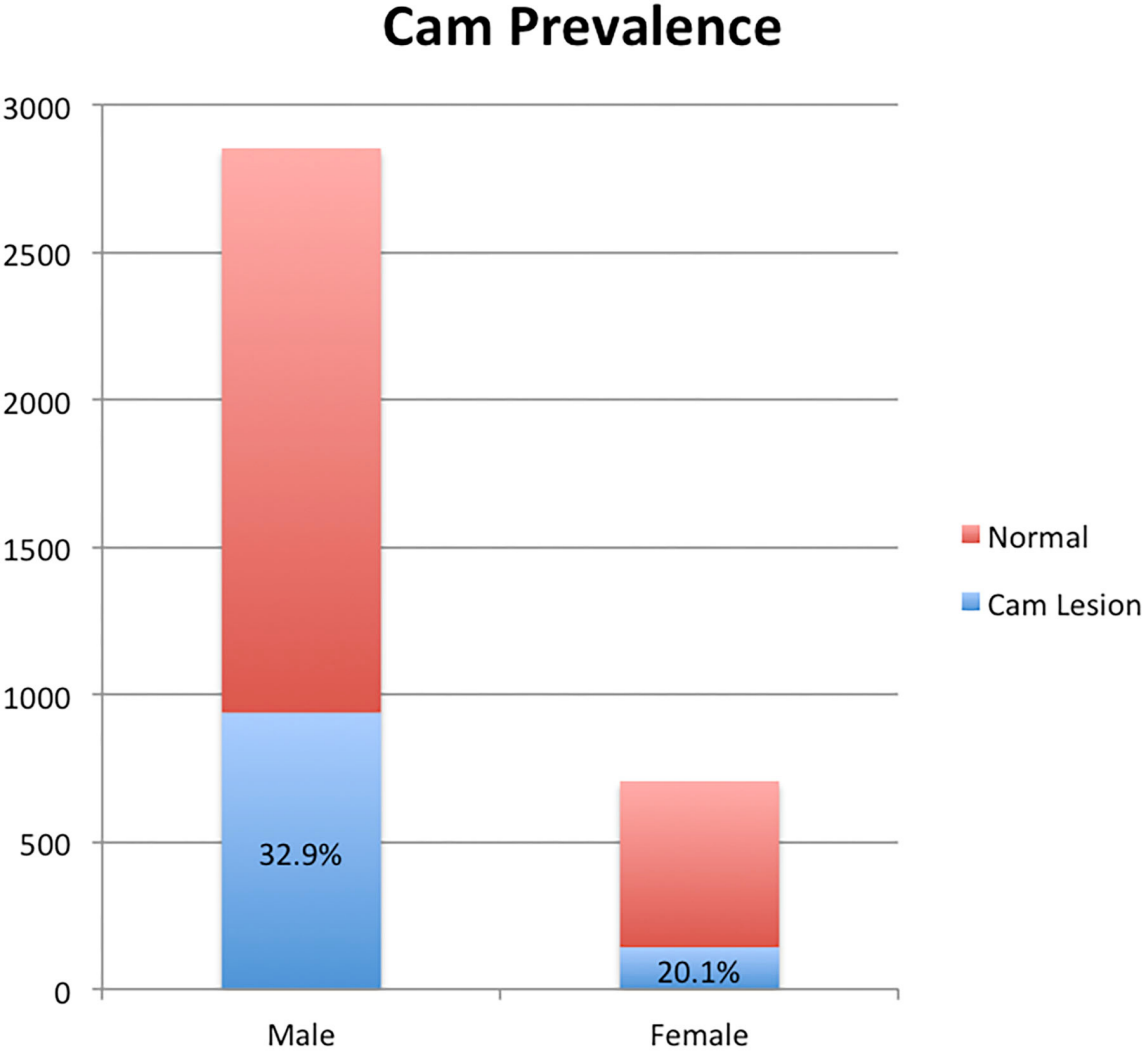
The prevalence of cam deformity witnessed in male and female specimens in the evaluated population.

Of the 3,558 femora measured, 2,852 (80%) were male and 706 (20%) were female. The overall prevalence of a cam deformity in our sample was 30% (1,080/3,558). The prevalence of cam deformity was 33% (938/2,852) in males and 20% (142/706) in females (Figure 4). A cam lesion was found in 30% (554/1,813) of right femora and in 30% (526/1,745) of left femora. We found bilateral cam deformities in 480 of 600 individuals who had a lesion on at least one side and a contralateral hip available for evaluation (80% bilateral prevalence).

### Reliability

The intraobserver and interobserver measurement reliability for the presence of cam deformity had weighted kappa values of 0.972 and 0.966, respectively.

### Location

**Figure 5:**
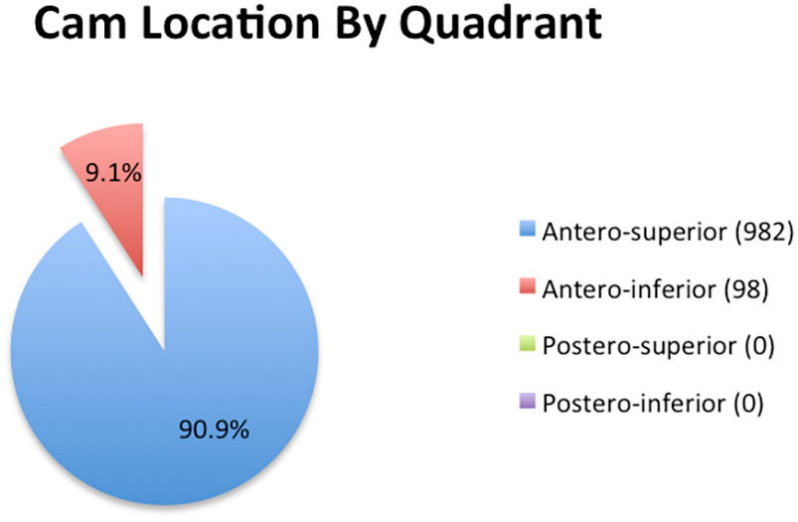
The location of cam lesions when stratified by quadrant.

Using our quadrant system, 982/1,080 femora (90.9%) were found to have a cam deformity in the anterosuperior quadrant (12:00–3:00). The remaining 98 femora had a deformity in the anteroinferior quadrant (9.1%). There were no maximal deformities in the posterior quadrants (Figure 5).

### Cam Deformity Size

For the male specimens: the average depth was 5.5 ± 2.2 mm; the average length was 16.9 ± 10.7 mm; the average width was 24.2 ± 11.9 mm. The largest cam deformity was 35 mm in length, 42 mm in width, and 12 mm in thickness.

**Figure 6:**
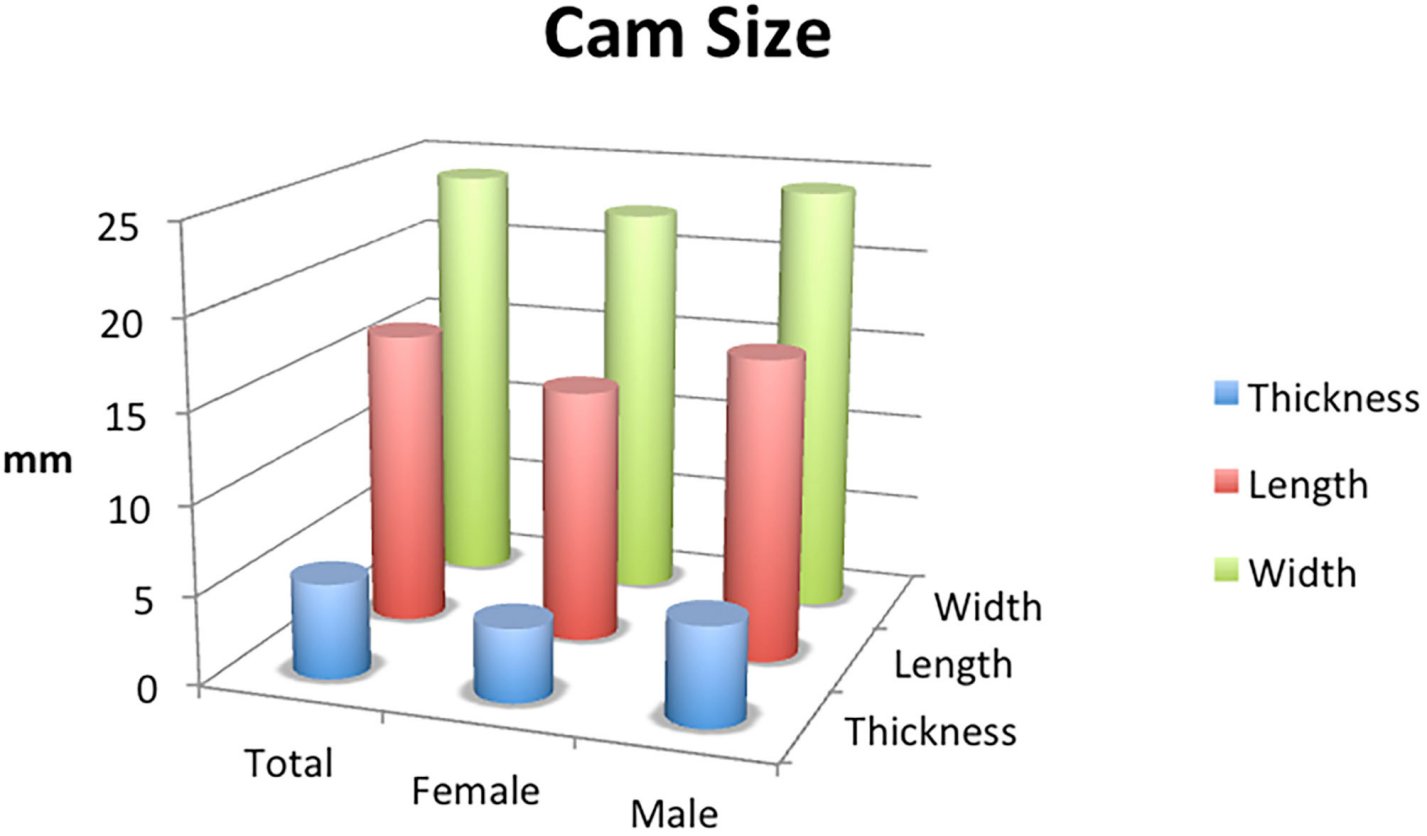
The average width, length, and thickness of cam lesions witnessed in male and female specimens in the evaluated population.

For the female specimens: the average depth was 4.1 ± 1.8 mm; the average length was 14.2 ± 8.9 mm; the average width was 22.3 ± 9.1 mm. The largest cam deformity was 32 mm in length, 38 mm in width, and 11 mm in thickness (Figure 6).

When comparing lesion size between men and women, there were statistically significant differences with regard to length, width, and depth (*p* < 0.05). There was no difference in size of the lesion based on laterality.

## Discussion

FAI has been established as an etiologic factor for the development of acetabular chondral lesions, labral tears, and eventual osteoarthritis of the hip (19). Therefore, the prevalence of FAI morphology may have direct bearing on the subsequent prevalence of these pathologies. Our study is the first to our knowledge to report cam prevalence based upon direct visualization in cadavers.

We have shown here that the prevalence of cam morphology of the proximal femur is higher than previously reported, with a prevalence of 26.5% (33% in men and 20% in women). Plain radiography, CT, and MRI have all been utilized to quantify cam prevalence. The Copenhagen Osteoarthritis Study reported a prevalence of cam morphology of 17% in men and 4% in women based upon alpha angle (>83° in males or >57° in females) and elevated triangular index (6) in 3,202 patients with AP pelvis radiographs (11). The prevalence reported in this study is lower than what we report; however, this study also had a few shortcomings. An isolated AP pelvis radiograph has poor sensitivity in diagnosing cam lesions and, therefore, it will underestimate cam prevalence. In a study comparing conventional radiographs to MR arthrogram, Dudda et al. (20) revealed that even when conventional radiographs were normal, there was often an increased alpha angle seen anterosuperiorly on MR arthrogram.

Given the shortcomings of plain radiography, other investigators have used three-dimensional imaging to ascertain cam prevalence. Tsitskaris et al. (14) reported cam prevalence of 19% (21% in men and 17% in women) in an asymptomatic population of 45 patients who underwent abdominopelvic CT for abdominal trauma or non-specific abdominal pain (based on an alpha angle >55°). Hack et al. (13) reported a prevalence of 14% (24.7% in men and 5.4% in women) using MRI (based upon an alpha angle >50.5°) in 200 asymptomatic volunteers (400 hips). Although the use of three-dimensional imaging is likely more reliable than the use of plain radiographs in ascertaining cam prevalence, it still has some shortcomings. Most of the studies reporting cam prevalence with three dimensional imaging rely on alpha angle to confirm the diagnosis. There has been broad variation in threshold values that have been used to define cam impingement (from 42 to 83°) (11, 21, 22). Additionally, Lohan et al. (17) demonstrated that the alpha angle has poor sensitivity and intraobserver reliability in diagnosing cam FAI in a study evaluating alpha angle determination on MR arthrogram by three fellowship-trained musculoskeletal radiologists (17). Specifically, the authors found that an alpha angle >55° had a mean sensitivity of just 39.3%, specificity of 70.1%, and poor intraobserver reliability (variations of as much as 30% in each observer). For this reason, the authors proposed the use of the AFD, which rendered improved sensitivity of 55.1%, specificity of 63.8%, and reliability. In light of the poor sensitivity and poor inter and intraobserver reliability of the alpha angle and wide range of alpha-angle thresholds used for diagnosis, alpha angle assessment on three-dimensional imaging is an imperfect way of determining cam prevalence.

In our evaluation, we found that 80% of patients with cam lesions had bilateral deformity. This figure is similar to data reported in published clinical series. Allen et al. (23) reported a prevalence of bilateral cam deformity of 77.8% (defined by an alpha angle >55.5°) in 113 patients seen in their office with hip pain. Of note, only 26% of patients with bilateral cam deformity (23/88) had pain in both hips, with an alpha angle of >60° being a significant predictor of having hip pain. Although this study specifically evaluated symptomatic patients with a known cam deformity, our results reinforce the high prevalence of bilateral cam deformity in the general population regardless of symptomatology.

Among those affected in our cohort, all cam lesions were witnessed in the anterior quadrants, with 90.9% in the anterosuperior quadrant and 9.1% in the anteroinferior quadrant. These findings are compatible with what has been reported in studies utilizing three-dimensional imaging to determine the location of cam lesions. Audenaert et al. (24) reported that the articular cartilage at risk was in the anterosuperior quadrant in each of 13 hips with cam lesions that were evaluated using CT with software segmentation. Reichenbach et al. (21) utilized radial MRI sequences to localize cam deformities by quadrant in 67 hips with “definite cam-type deformities.” Among the 67 deformities, 61 (91%) were located in the anterosuperior quadrant, three (4.5%) were in the anteroinferior quadrant, two were posteroinferior, and one was posterosuperior.

Our study has several strengths. We sampled a large cohort of the regional Cleveland population, selected the age group most commonly affected by FAI (individuals 18–55 years of age), and did not specifically select individuals with the presence or absence of hip symptomatology, which allowed us to most accurately estimate the overall cam prevalence in the regional population. Our study also has several limitations. A primary shortcoming of our study is the use of a non-validated method of determining the presence of cam deformity (AFD > 2 mm) instead of the alpha angle. Nevertheless, when this method was employed radiographically by Lohan et al. (17), they witnessed improved intraobserver reliability and improved sensitivity, positive predictive value, and negative predictive value of detecting an arthroscopically significant cam lesion. Likewise, we witnessed strong interobserver and intraobserver reliability using this method (weighted kappa values of 0.972 and 0.966, respectively). This study is also limited by selection bias as the Hamann–Todd collection is disproportionately composed of specimens who were male (80% male), died at a younger age, African-American (half of the specimens in the collection are African-American, and half were Caucasian with no inclusion of other races), and from low socioeconomic strata (25). The prevalence of cam deformity in our population may not be broadly generalizable to the modern American population due to regional variations in genetics and temporal variations in environmental factors (in light of more recent increases in popularity of sports linked to cam development). Additionally, in light of male predominance of our sample and the male predilection for cam deformity, the 30% prevalence that we report likely overstates the proportion of the population with cam deformity. The estimated prevalence in the population at large is likely the average between the male prevalence and female prevalence, or 26.5%. Furthermore, femora with significant osteophytes based on inspection were excluded from the study, but femora with small osteophytes may have been confused as a cam deformity and included in the analysis. Finally, although our technique effectively captures cases of cam deformity secondary to femoral head asphericity, our method likely misses cases of head–neck offset and femoral retroversion.

Our study reports the prevalence of osseous cam deformity in a cross section of a historic general population from a large urban area. It suggests that the prevalence of a cam deformity of the femoral head–neck junction may be significantly higher than has been previously reported. Previous studies estimating cam prevalence based upon imaging may have underestimated the true prevalence due to the poor sensitivity of the AP pelvis radiograph in capturing more anteriorly located lesions and the poor reliability of the alpha angle in diagnosing cam lesions. Our series represents the first study to quantify cam prevalence based on direct visual anatomical inspection. This series provides surgeons with a firm baseline for the prevalence of cam deformity, prevalence of bilateral cam deformity, location of cam lesions, and the typical dimensions of cam lesions. We are aware that these findings are not directly transferrable to clinical practice, since the diagnosis of a cam deformity contributing to FAI is generally based on the combination of symptoms, clinical findings, and radiologic findings that also include soft tissue pathology. The clinical setting does not afford the possibility of direct visual assessment of the osseous femoral head–neck junction and, therefore, must rely on radiologic parameters. Thus, we cannot offer any specific recommendations regarding treatment strategies with regard to extent of resection. Nevertheless, we believe that our findings are relevant to a clinical audience since they raise awareness regarding a high osseous prevalence of cam lesions and focus attention to specific areas of the femoral head–neck junction. As the correlation of FAI with the development and causation of hip osteoarthritis is further investigated, our findings provide some useful baseline information with regard to the prevalence and location of osseous deformity at the head–neck junction.

## Data Availability Statement

The raw data supporting the conclusions of this article will be made available by the authors, without undue reservation.

## Author Contributions

All authors made significant contributions to study design and conception as well as manuscript preparation.

## Conflict of Interest

The authors declare that the research was conducted in the absence of any commercial or financial relationships that could be construed as a potential conflict of interest.
